# Co-production in practice: how people with assisted living needs can help design and evolve technologies and services

**DOI:** 10.1186/s13012-015-0271-8

**Published:** 2015-05-26

**Authors:** Joseph Wherton, Paul Sugarhood, Rob Procter, Sue Hinder, Trisha Greenhalgh

**Affiliations:** Centre for Primary Care and Public Health, Barts and the London School of Medicine and Dentistry, Yvonne Carter Building, 58 Turner St, Whitechapel London, E1 2AB UK; East London NHS Foundation Trust, London, E1 8DE UK; Department of Computer Science, University of Warwick, Coventry, CV4 7AL UK; Department of Primary Care Health Sciences, University of Oxford, 2nd floor, New Radcliffe House, Walton St, Oxford, OX2 6GG UK

**Keywords:** Assistive technology, Ethnography, Co-design, Co-production, Telehealth, Telecare

## Abstract

**Background:**

The low uptake of telecare and telehealth services by older people may be explained by the limited involvement of users in the design. If the ambition of ‘care closer to home’ is to be realised, then industry, health and social care providers must evolve ways to work with older people to co-produce useful and useable solutions.

**Method:**

We conducted 10 co-design workshops with users of telehealth and telecare, their carers, service providers and technology suppliers. Using vignettes developed from in-depth ethnographic case studies, we explored participants’ perspectives on the design features of technologies and services to enable and facilitate the co-production of new care solutions. Workshop discussions were audio recorded, transcribed and analysed thematically.

**Results:**

Analysis revealed four main themes. First, there is a need to raise awareness and provide information to potential users of assisted living technologies (ALTs). Second, technologies must be highly customisable and adaptable to accommodate the multiple and changing needs of different users. Third, the service must align closely with the individual’s wider social support network. Finally, the service must support a high degree of information sharing and coordination.

**Conclusions:**

The case vignettes within inclusive and democratic co-design workshops provided a powerful means for ALT users and their carers to contribute, along with other stakeholders, to technology and service design. The workshops identified a need to focus attention on supporting the social processes that facilitate the collective efforts of formal and informal care networks in ALT delivery and use.

## Introduction

### Telecare and telehealth

An ageing population is fuelling interest in assisted living technologies (ALTs), including *telecare* (remote monitoring of emergencies through sensor devices and personal alarms) and *telehealth* (transmission of medical information over telecommunication). There is much policy investment in delivering these services on a large scale. For example, the Whole System Demonstrator (WSD), the largest randomised controlled trial of telecare and telehealth to date, was conducted in England in 2008–2011 to provide evidence for cost-effectiveness. Disappointingly for policymakers, the intervention had no significant effect on care efficacy and did not reduce costs to services [1, 2].

Despite continuing debate about the significance of the WSD [3–5], investment has continued into large-scale initiatives [6]. This has been driven by a modernist vision of ALTs as inevitably useful, empowering and unobtrusive, and a pro-innovation bias for the widespread deployment of new technologies to increase service efficiencies and cost savings [7].

In the Assistive Technologies for Healthy Living in Elders: Needs Assessment by Ethnography (ATHENE) study, we conducted ethnographic research that illuminated the complex living experiences and needs of 40 people aged 60–98. We visited participants at home several times and used narrative interviews, home tours and cultural probes to build a detailed picture of participants’ lives, illness and use (or non-use) of technologies. The cultural probe method applies everyday artefacts and tools for participants to use in their own time to help depict their lives to researchers [8]. We developed a version of the cultural probe, the ‘Home and Life Scrapbook’, which consisted of a digital camera and open-ended activities (e.g. a ‘wish list’, ‘relationship map’) to capture information on physical, emotional and social factors related to health and independence [9].

We produced a rich case narrative for each participant, covering their experience of ageing and ill health, what mattered to them, and use (or non-use) of technologies. The analysis is described in detail in other publications [10, 11]. In addition, 23 participants consented for their case summary to be published on the ATHENE website [12].

Analysis of case studies showed that participants’ needs were diverse and unique, and that ALTs were rarely fit for purpose. However, we also observed some successful technology arrangements. These were characterised by ‘bricolage’, in which relatives of an older person (and sometimes the older person themselves) adapted existing technologies very creatively and effectively to produce a ‘personalised’ solution. Unfortunately, neither the design of the technologies, nor the services that use them, acknowledge the need to take active steps to promote bricolage. If ALTs are to be useful and sustainable, their role must be understood as elements of a socio-technical infrastructure that need to be designed and deployed in ways that are compatible with the social relations that make them ‘work’. Technology suppliers and service providers need to rethink how they can work with older users and their networks of carers to co-design and co-produce useful and useable solutions.

### Technology co-design workshops

Methods from the participatory design tradition are increasingly being seen as providing a strategy for patient involvement in health service improvement [13]. Robert and colleagues have developed the science of ‘experience based co-design’ to bring about patient-led clinical service improvements [14, 15]. Their approach entails staff, patients and carers reflecting on their experiences of a service to identify improvement priorities and devising changes. Storytelling of memorable ‘touch points’ with the service provide a focus for communicating all aspects of the patient and carer experience.

Co-design workshops are widely used in participatory design to help users and designers express and exchange ideas [16, 17]. Robert et al. have used such workshops extensively in service redesign efforts [14, 15]. In a more technology-focused tradition, a similar application of co-design principles aims to ensure that technologies *and* the services in which they are embedded co-evolve in a way that is grounded in the lived experience of users, who are fully engaged in the design process [18].

Previous authors have reported that involving older people in co-design has been challenging as a result of sensory impairment, cognitive difficulties, mobility needs, fatigue, and lack of technical knowledge [19–21]. Techniques developed to help engage older people more democratically in the co-design process have included use of visual aids and interactive tasks (e.g. card prompts, task-flow diagrams) to focus attention on specific aspects of a technology design [22–25]. User narratives such as stories and scenarios may be used in co-design to communicate design concepts and envision how they might be used [26–28].

This paper reports our use of co-design workshops to bring ALT users and stakeholders together to discuss the ATHENE ethnography and their views on technology and service design. The aim was to establish the requirements of a socio-technical infrastructure to support co-production. We did not have any particular technologies or services in mind but wanted to elicit the priority concerns and design ideas from users and providers of ALTs. To this end, the workshops were structured to encourage participants to lead discussion on technical, social and organisational factors.

## Methods

### Sample

Ten workshops were conducted with a total of 61 participants. Four were held with a total of 30 end-user representatives, all of whom had some experience with using, or helping someone to use, telecare and/or telehealth. This included participants from the ATHENE ethnography, their informal carers, and third-sector advocates. They represented a range of health conditions (e.g. chronic obstructive pulmonary disease, diabetes, heart disease), physical and sensory impairments and ethnic backgrounds. Three participants spoke limited English, so an interpreter was present during the workshops. Participants varied greatly with regard to use of ALTs, in which some were active users and others were unable to (or decided not to) use the technology.

Three workshops were held with 18 service provider representatives, including occupational therapists (OTs), nurses, monitoring centre operators, technicians, managers and commissioners involved in the provision of telecare or telehealth. Two workshops were held with 13 technology industry representatives, including designers, engineers, software developers, business analysts, and sales and marketing staff.

The final workshop brought together 11 representatives from across these different user and stakeholder groups; it included two ALT users, two technology industry and seven service provider representatives. All workshops lasted approximately 2 h.

Ethical approval was gained from Queen Mary University of London Research Ethics Committee and the NHS Research Ethics Committee. The ALT users were invited to the workshops and asked to invite others who help them. The industry and service representatives were recruited via networking events on assisted living and our steering group. All participants consented to be audio recorded. We have deliberately not given detailed information about participants or organisations to preserve anonymity.

### Co-design workshops

**Fig. 1:**
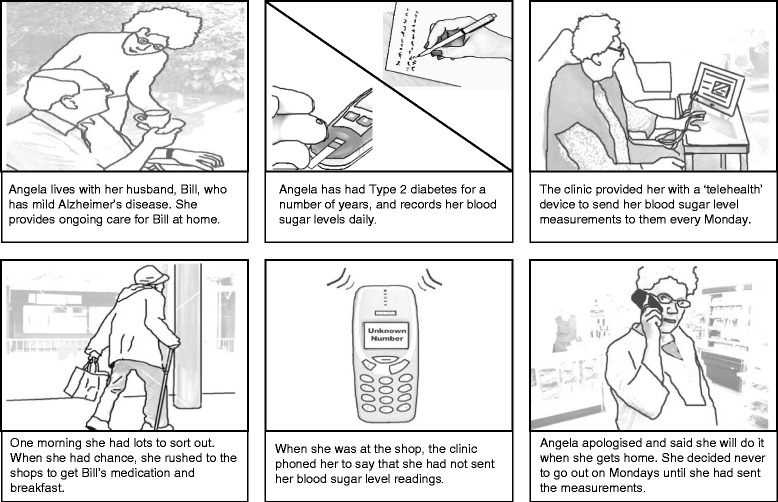
Example of ‘cartoon strip’ approach to generating discussion about case scenarios. In this example, *Frame 1* introduces the characters (Angela who is wife and carer for her husband, Bill, who has mild dementia). *Frame 2* explains that Angela has diabetes and records her blood-sugar levels daily. *Frame 3* describes the introduction of telehealth for Angela to send her blood-sugar readings to a clinic on a weekly basis. In *Frame 4*, Angela rushes to the shops to get Bill’s medication and food for breakfast. In *Frame 5*, the clinic calls Angela on her mobile phone while she is at the shop because she forgot to send her blood sugar readings that morning. In *Frame 6*, Angela apologises for forgetting to send her measurements through the telehealth unit and informs the caller that she will do it once she gets home

In the four end-user workshops, vignettes were presented using ‘storyboards’, which depicted (in cartoon-strip format) a narrative. The stories were fictional but based on real accounts of problems encountered with various ALTs. Each storyboard consisted of six frames that were structured to introduce the characters/setting, the person’s care needs, the technology(ies) in place, a problem with the technology and response to the event (Fig. 1). Facilitators presented the storyboard before opening up for discussion with the group.

A total of six storyboards were produced, with three presented at each workshop. Scenarios were based on recurring themes from the ethnography. Figure 1 depicts how services for technologies made high (and sometimes oppressive) demands on users. Other scenarios included issues related to usability, fitting technology into domestic settings and anxieties around use.

The second part of the workshop focused on ALT devices and service provision. The focus on technology was facilitated using card prompts depicting different devices or design features. Participants had their own deck of cards that they were asked to select and arrange into ‘most useful’ and ‘least useful’. For example, one deck of cards represented telehealth features to support self-management (feedback displays, educational videos, motivational message, reminders, etc.). A blank ‘something else’ card was included to invite alternatives. Discussion on service design was facilitated by a flow diagram representing stages in ALT provision (‘assessment’, ‘decision for telecare/telehealth’, ‘installation and training’ and ‘review’) to focus on their experiences or concerns at each stage.

The service provider and technology industry workshops included anonymised case narratives and extracts (quotes, cultural probes, photos) from the ATHENE ethnography. Participants were sent the case summary prior to each workshop (with participant’s consent) and asked to reflect on three questions: (a) Bearing in mind what matters to this person(s), how could their life be improved through a technology or service? (b) What would be the issues/challenges implementing one of these solutions and how might these be overcome? and (c) How might the technology or service be sustained and adapted over time?

Following discussions about the vignettes, the service provider workshops focused on implications for service design (facilitated by a flow-diagram of the service delivery process, such as ‘assessment’ and ‘review’), and industry workshops centred on implications for technology development (facilitated by presentation of design terms, such as ‘requirements gathering’, ‘prototyping’, ‘user feedback’).

Workshops were audio recorded, transcribed and analysed using constant comparative analysis [29]. Transcripts were first broken down into concepts and organised to summarise key issues from each workshop. All authors reviewed and discussed concepts immediately after each workshop so that emerging issues could be explored in subsequent workshops. Data were then combined to provide a summary of emerging themes for end-user, service provider and industry workshops, which were used to narrow focus for the final cross-sector workshop. The cross-sector workshop was focused around an anonymised case summary to facilitate discussion on particular issues. The need for knowledge sharing and coordination across services occurred frequently across all workshops. Therefore, discussion on this issue was supported using prompt cards to ask the different representatives to think about how they would like to communicate with each other (e.g. What information would be useful? Who would you like to communicate with?). Again, this workshop was analysed as described above to inform and refine the main themes.

## Results

All 10 workshops engaged participants in a lively and creative forum in which they discussed the ethnographic data and generated numerous technology and service design ideas. Analysis revealed four main themes: (a) raising awareness and sharing knowledge; (b) customisation and adaptation; (c) ongoing social support and (d) information sharing and coordination. We consider these in turn below.

### Raising awareness and sharing knowledge

The need to increase public awareness about ALTs recurred frequently. Users and their carers were motivated to explore how the technology could support them but felt restricted by lack of information and guidance. Similarly, service providers and industry representatives called for greater efforts to increase public awareness and understanding of ALTs. They identified a need for more training of care staff to improve patient signposting and assistance.

End-user representatives also talked about the value of learning through direct interactions with other people using or supporting the use of ALTs. This was evident during co-design workshops, in which they shared knowledge and strategies related to specific problems. This led them to conclude that social gatherings or ‘forums’ would help them take initiative to resolve problems and innovate solutions:

Word of mouth is the best way, when you’ve met someone, they say there’s this or that. A lot of it is being in the right place at the right time. Finding out little snippets of information, picking people’s brains. [Garry, son and carer for mother, Molly aged 77]

Similarly, service provider and industry workshops identified a need to support ‘shared learning’ across services and sectors about the capabilities and limitations of technology, and workarounds to problems:

Shared learning is needed. With daily living equipment, we’ve been through that over many years, so we know which pieces of equipment work well and we share that as a group. But that hasn’t happened the same way on the technology side. [Service Provider, OT]

For many service providers, their encounters with industry had centred on the promotion or selling of products, rather than mutual learning:

We’d love to speak with industry more. Not from a sales perspective…It’s funny, because if I say, ‘I might want one or two on a trial’, they’re not interested. They only want to know if we want about 300. [Service Provider, OT]

### Customisation and adaptation

The ALT users described how they experienced technologies that had not been personalised as disruptive and stressed that, to be acceptable, ALTs must offer sufficient flexibility to fit into everyday life and fluctuations in their capabilities and routines.

The card selection activities provoked discussion among users, who proposed different ways that they would adapt and use the technology. These ideas drew on knowledge of their own capabilities, environments and risks. For example, one user with diabetes was anxious that she would not be able to find her pendant alarm in the dark during the night. Her blood sugar level tended to drop at night, but she often forgot where she put her pendant. She discussed with the workshop group the possibility of mounting a large button by her bedside.

In another example, a participant with visual impairment felt that she would benefit from a series of pull-cord alarms located en route from her bedroom to the bathroom. Her main concern was risk of falling during visits to the bathroom, but (perhaps because of mild cognitive impairment) she rarely remembered to carry her pendant alarm with her at this time. A third telecare user was more concerned about falling outside the home (following a recent fall in a car park), and so wanted her pendant alarm to work outdoors. These examples illustrate how different users proposed different ways to configure and use the same, and most basic, form of telecare—personal trigger alarms. In fact, all three of the configurations proposed by these participants are already possible with existing telecare equipment (wireless large button triggers, pull-cords, mobile telecare with GPS tracking, respectively). However, all three were unaware of the relevant technology and were making do with the standard telecare package that had been supplied to them.

Care professionals whose role was to assess for and implement ALTs discussed the constraints to personalisation. First, they talked about the effortful task of initial assessment, which was considered critical to getting to know the specifics of how the user lives and experiences their illness. Failure to do this effectively could actually worsen, rather than improve, the situation for the older person, and so was seen as an ethical as well as a practical issue:

People think, if you’ve got the technology in, it will at least help. But actually if you don’t get the detail, the crux lies in the details, if you don’t get it right, then it can potentially be detrimental, even small things. [Service provider, OT]

Second, care professionals’ accounts of installing ALTs in users’ homes illustrated that this task requires considerable hands-on, practical reasoning to fit the technology around the individual contexts, material constraints and the particular ends that are to be achieved:

We tend to think ‘I need it quickly’, rather than thinking wider…Sometimes we’ve tried to be really creative. And to be honest, it doesn’t always work, because the kit is designed for a certain function, when you try and adapt it. We have to do it on a trial basis. [Service provider, Telecare Lead]

Care professionals also felt that the importance of material knowledge and practical reasoning was not fully appreciated across the services. Staff often did not have the time or experience to adapt solutions in this way, and patients tended to be passed through distinct care teams, each with specific responsibilities and tasks, along a so-called ‘care pathway’—and installing technology tended to be viewed as a one-off technical procedure in this pathway rather than a more organic customisation process.

Service provider and industry representatives talked about the restricted range of technology options that may be implemented for a particular user due to contractual limits on what could be provided by whom. Service staff talked of being ‘locked in’ to particular suppliers, determined by commissioning decisions:

The equipment was bought by commissioners, non-clinicians. Had there been more engagement with clinicians who had an understanding of the patients who use it, it might be slightly different. [Service Provider, Commissioner]

Technology developers highlighted current technical barriers to customisation. This includes a lack of interoperability across ALT suppliers, which limits the extent to which devices can be combined in accordance with the user’s needs. A second technical constraint related to the safety and reliability of ALTs that were open to ongoing configuration:

I think the issue of bolting on a device that you’ve picked up in a shop, that’s where it becomes quite tricky. Because you’ve got, what we class as a safety critical device. [Industry, Marketing Lead]

### Ongoing social support

Participants talked about the role of informal social support (e.g. family, friends) to help introduce ALTs and overcome technical glitches or limitations. Our data showed that users’ informal resources varied greatly. For users who depended solely on professional services, even minor problems (e.g. replacing batteries on a device) could pose significant disruption:

I heard this beep, beep…It wanted a battery, all it needed was a battery. But I didn’t know where the battery was to go…It took them so long to come and change the battery for me....It was about two weeks before Christmas that I told them, and it went through Christmas and then New Year and then it was January before they came. [Elsie, aged 82]

Workshop participants identified a need for service providers to assess the older person’s informal support and align the ALTs deployed with these resources. In addition, they felt, the service would require capacity to respond to diverse (and sometimes ‘minor’) needs when such resources were not available. Service users emphasised that staff should reach out to users to support them with technology and create a sense of familiarity and presence.

An important part of providing this support, end-users felt, was to build positive personal relationships with the ALT user and their carers. They emphasised that regular contact and on-hand support was particularly important at the early stages of using the technology. They felt that this investment of human effort would ultimately lead to more effective solutions:

If you called in perhaps the next day or a couple of days later, had a cup of tea and talked it over, you’d find where the difficulties are…And that second or third visit to see would make all the difference. [Mrs K, aged 80]

Service providers recognised that relationship formation was important for effective ALT implementation and use. Getting to know individuals over time surfaces the subtle, granular information about their life (e.g. illness, events, anxieties) that have a bearing on the appropriateness and use of the technology. Call centre staff were seen to play a particularly important role in this regard through frequent and opportunistic interactions with clients, which helped them interpret and respond to remote monitoring information in context:

We recently had a patient that’s going through a divorce. She’s quite weepy, she’s stressed. So it’s learning that information. We aren’t just looking at the readings and saying, well, that’s high. [Service Provider, Telehealth monitoring operator]

Workshop participants concluded that services should invest time and effort into maintaining frequent contact with clients who needed this, and exploit opportunities for interaction. In practice, such interactions currently occur on an ad hoc basis and are treated as aberrations of usage (e.g. ‘false’ alarms or clients triggering alarms ‘for a chat’). They felt that services should be designed to facilitate informal and interpersonal interaction as a component of routine practice. Additionally, technical subsystems might even be designed to prompt and encourage interpersonal interaction in order to develop a positive personal relationship with the service when desired. Rather than developing technologies to become more ‘independent’ from the care network, design should focus on the social cohesion required to support the older person alongside ALTs:

We have to be aware that we don’t get in the way of social networks…Maybe the answer isn’t to make technology as simple as possible. Maybe the answer is to make it as socially adaptive as possible. [Industry, Business Analyst]

### Information sharing and coordination

A recurring theme was the need to support knowledge sharing and coordination within and between services, as well as across formal and informal care networks. Service chains—with several people involved in supporting an individual—are complex and tend to lack effective integration. Aspects of the ALT service (e.g. installation, monitoring) are often outsourced to subcontractors, which adds another level of separation.

The workshops suggested that substantial improvements in intra- and inter-agency coordination and information sharing is needed to provide a holistic view of the older person and track changes in circumstances and needs. Participants identified a potential role of ICT to support information sharing and coordinated activity. Their suggestions included features to increase awareness of other care activities, tracking of technical issues and a directory to seek expertise to resolve problems:

Knowing that a district nurse is planning a review, we could slot in a couple of questions, or catch up with that nurse afterwards, or share that information. I just don’t think we do enough. [Service provider, Community Matron]

These suggestions by participants align closely with findings from the computer supported cooperative work (CSCW) literature that social and technical subsystems should be organised to support collaboration through mutual awareness (the sense of what the other collaborators are doing in order to provide a context for your own activity) [30] and facilitate sharing of both ‘formal’ knowledge (documented and accessible by people within an organisation) and ‘informal’ knowledge (gained through everyday practice that is not documented) [31].

However, participants also identified that such a platform would need to accommodate multiple actors across the care network. Different people will hold different roles, knowledge and expertise, and so the design would require multiple representations that are attuned to the particular goals and tasks of each member. In addition, they felt that these representations should include means to access day-to-day knowledge and experience within the informal network:

I think if you gave an opportunity to put something on themselves. When you get it in their own words, you get much more of a flavour of how they actually see it. [Service Provider, Community Matron]

## Discussion

Ethnographic data was taken forward through co-design workshops to facilitate discussion about the design of ALTs and services that support their use. Four major needs were consistently raised: (a) raising awareness and sharing practice knowledge; (b) customisation and adaptation; (c) ongoing social support and (d) information sharing and coordination among professional carers.

### Raising awareness and sharing practice knowledge

Lack of awareness among users and services has long been considered to be one of the main reasons for poor uptake of ALTs [32, 33]. Numerous information resources have been developed [34–36], but there remains a need for more effective methods for providing information and guidance [37].

Lave and Wenger [38] introduced the concept of ‘communities of practice’ to describe how groups share information and learn from each other. Such groups often evolve naturally through a common interest, to share experiences and validate knowledge. This not only helps develop members’ explicit knowledge (knowing what), but also their tacit knowledge (knowing how). Brown and Duguid [39] applied this concept to technical work. Drawing on Orr’s [40] ethnographic studies of photocopier technicians, they showed how practitioners who install and ‘fix’ technologies pass on practical wisdom (essential to supplement the standard operating procedures of official manuals) through personal interaction and storytelling, for which informal contact and friendship-building is crucial.

Communities of practice have emerged in a range of care services, with much diversity in how and why they developed [41]. However, by their very nature, communities of practice are informal and unstructured, and so difficult to establish. A further complexity for telecare and telehealth lies in the inter-sectoral links between public and industry sectors, characterised by contractual agreements and multiple perspectives and interests. Nevertheless, there is much scope for encouraging the role of knowledge brokers in the public, private and third sectors to raise public awareness and provide information to potential users of ALTs and their carers so that they can take initiative to devise solutions and perform bricolage effectively.

### Customisation and adaptation

Participants’ discussions about the technology focused on the need for greater configurability, as opposed to new devices or design features. Customisation and adaptation of ALTs raises a number of challenges. Not only does this imply that individual devices be designed with configuration in mind, but also that they be capable of being assembled into larger configurations. This would enable, for example, different monitoring devices to integrate with a single unit for the transmission of readings. Moreover, such a configuration could be adapted (e.g. addition of new devices) as the person’s needs change. To achieve this, ALTs must be designed as composable units that enable straightforward construction of more complex and bespoke solutions [11, 42].

To produce technologies that are highly personalisable, the ALT industry will have to address the tension between commercial rivalries and the ideal of inter-operability. In the UK, despite the efforts of the Continua Alliance [43] to promote interoperability standards among suppliers, progress towards this goal is slow. Such a fundamental reconfiguration of the mode of technology supply may take time to achieve and will be resisted when suppliers’ business models rely on ‘locking’ users into their products.

However, it is important to acknowledge industry’s legitimate concerns that customisable and adaptable solutions would place greater demand on dependability mechanisms to ensure and maintain system reliability and integrity. Increasing customisability of devices also raises the question of the extent to which industry may be held responsible for the consequences of modifications or alterations by users and their carers.

### Ongoing social support

Installation of ALTs must cease to be a one-off *technical* event and become an ongoing process of *personal and social* support built through persistent relationships and social networks. Participants’ accounts highlight the importance of interpersonal qualities of service staff and relationship formation with users and their informal carers. Success of a solution and capacity to collaboratively adapt solutions through bricolage depends crucially on familiarity and trust—yet some service configurations militate against this. For example, design efforts towards cost-effectiveness and ‘scalability’ (e.g. centralisation of telemonitoring centres, automated devices) could inhibit the human relationships that make the technologies ‘work’. Relationship formation has become an established component of holistic nursing care [44, 45]. Research into social dynamics in ALT implementation could inform how to enhance these relationships to support co-production.

A second interpersonal challenge is the motivation across multiple actors to engage in the collective task of supporting the older person over time. ALT users emphasised the role of family and friends to perform a range of ad hoc and often ‘minor’ tasks to support use of the technology (e.g. replacing batteries, repeatedly demonstrating how to use devices), and felt that the services should take on such roles in the absence of sufficient informal resources. However, it is difficult to specify these tasks and roles in advance. Services would require a collegial and altruistic motivation to engage, as opposed to contractual protocols and conditions. Pro-sociality (going beyond formal job requirements and procedures) is often seen as an integral part of the job within healthcare because the precise combination and sequence of skills required to treat a patient are often difficult to anticipate [46]. In healthcare settings, frontline staff have first-hand contact with patients and so have an advantage in establishing the empathy that motivates pro-sociality. A challenge for telecare and telehealth will be to foster a similar understanding across individuals who contribute to the implementation and sustained use of the technology, in the absence of prolonged engagement with the ALT users.

### Information sharing and coordination among professional carers

As well as increasing explicit and tacit knowledge about ALTs (discussed above), workshops identified a need to support information sharing about the needs of a user. Contributors to the co-production and bricolage of personalised ALT supports need to harness and share knowledge about the older person and the solution effectively. It is therefore quite timely that there has been growing technical attention to the integration of health data and social computing [47, 48]. However, these systems are designed with a strong emphasis on privacy and accessibility of patient data, with little focus on the social dimension. The workshop participants’ views align with the CSCW notion of ‘common information space’, which has been influential in how we think about collective and coordinated activity [49]. The workshops identified ‘social affordances’ (properties of technology or environments that permit social interactions) [50] to support co-production. First, participants identified a need to facilitate both ‘formal’ and ‘informal’ knowledge sharing about the older person. Current methods, or ‘workarounds’, to achieve this have been through effortful and opportunistic actions to seek and utilise information that is not routinely captured or shared (e.g. user experiencing a health problem or physical impairment). Second, participants identified a need to enhance mutual awareness—the sense of what the other collaborators are doing. This is particularly important in the context of assisted living, with continually changing care needs (e.g. a change in care support package).

Studies have shown that ICT can offer support for coordinated activity through multiple representations of the same underlying information about a patient. Unlike paper documents, electronic records can offer different interfaces and ‘views’ so that it is more attuned to what each person needs to know. Such ‘de-coupling’ of information from its representation has been found to be effective in supporting inter-professional coordination within hospital settings [51], and so it is possible that a shared technical platform could also help coordination across a distributed care network. For example, workshop participants discussed how ICTs could represent information about the older person that was relevant to the different people in their care network (GPs, care workers, telecare monitors, etc.). Additionally, they discussed how ICTs could help bridge the gap between the clinical and experiential representations by allowing patients and their carers to input and share their own accounts. However, if this is to work, several challenges will have to be met. For example, it will be important to establish the extent to which such information can be re-represented, without loss of meaning. Furthermore, both lay and professional carers may be reluctant to place ‘sensitive’ information on a shared platform.

Most important of all will be to ensure that any technological intervention affords social interaction rather than getting in its way. In sum, the aim must not be to make ALTs independent of social networks but to align the technologies with them.

### Conducting co-design workshops

We have found that vignettes from ethnographic data facilitate the direct involvement of older people in technology and service design. The storyboards provided users and their carers with a ‘common ground’ [52] to facilitate communication amongst people with different knowledge and experience with ALTs. The inherent structure of the story provoked dialogue about how problems should be resolved and the frame-by-frame format allowed participants to pick-out certain aspects of the scenario to discuss.

Similarly, the anonymised case summaries were important during stakeholder workshops, as they helped disrupt prior assumptions about how the user interacts with the technology and service. Despite their routine encounters with users, service providers commented how the narratives helped them think differently about ALT provision. Likewise, the technology developers commented how the ethnographic accounts enabled them to relate their technical knowledge of the solution to the lived reality of the user and the service in which it is embedded, as opposed to reviewing the technology design in isolation. Developers and providers of ALTs need to devise ways to routinely capture such detail in their work practice. A major challenge here will be to move from a labour-intensive, ‘research quality’ assessment of people’s lives and lifestyles to a briefer but still effective (and cost-effective) approach.

Whereas vignettes supported discussion around contextual factors, the card prompts and flow diagrams focused attention towards specific technical and service design features. For example, the visual arrangement of participants’ cards allowed them to view and compare each other’s decisions, and think about how the technologies could be tailored to their particular needs. In this study, their ideas were confined to their discussions and annotations of card prompt materials. Future work should explore the role of similar collaborative formats to support service engagement with users in order to devise more personalised and effective solutions.

## Conclusions

Participants identified technical and social challenges to supporting bricolage and co-production. They identified that technologies must be customisable and adaptable but maintain system integrity. In addition, services must be aligned with the individual’s support network and provide a high degree of information sharing to track their needs over time. The ALT community needs to shift focus from the development of advanced technologies and deployment at scale to looking at the technical and organisational change required for their introduction and supported use.

## Abbreviations

ALT: assisted living technology
NHS: National Health Service
